# Dysregulation of microRNA expression during the progression of colorectal tumors

**DOI:** 10.1111/pin.12975

**Published:** 2020-06-26

**Authors:** Makoto Eizuka, Mitsumasa Osakabe, Ayaka Sato, Yasuko Fujita, Yoshihito Tanaka, Koki Otsuka, Akira Sasaki, Takayuki Matsumoto, Hiromu Suzuki, Tamotsu Sugai

**Affiliations:** ^1^ Department of Molecular Diagnostic Pathology, School of Medicine Iwate Medical University Iwate Japan; ^2^ Department of Surgery, School of Medicine Iwate Medical University Iwate Japan; ^3^ Division of Gastroenterology, Department of Internal Medicine Iwate Medical University Iwate Japan; ^4^ Department of Molecular Biology, School of Medicine Sapporo Medical University Hokkaido Japan

**Keywords:** colorectal adenoma, colorectal cancer, intramucosal carcinoma, microRNA

## Abstract

MicroRNAs (miRNAs) are potential biomarkers of neoplastic lesions, but additional information on dysregulated miRNA expression during progression of the adenoma–adenocarcinoma sequence may be helpful to identify the role of miRNAs in this sequence. We examined the expression levels of 13 miRNAs (hsa‐miRNA‐19a‐3p, hsa‐miRNA‐21‐5p, hsa‐miRNA‐27a‐3p, hsa‐miRNA‐27b‐3p, hsa‐miRNA‐31‐5p, hsa‐miRNA‐34b‐3p, hsa‐miRNA‐125b‐5p, hsa‐miRNA‐143‐3p, miRNA‐191‐5p, hsa‐miRNA‐193b‐3p, hsa‐miRNA‐195‐5p, hsa‐miRNA‐206 and hsa‐let‐7a‐5p) that are closely associated with colorectal carcinogenesis in 40 conventional adenomas (tubular and tubulovillous adenomas), 20 intramucosal carcinomas (IMCs) and 60 invasive colorectal cancers (iCRCs) using reverse‐transcription polymerase chain reaction. These 120 tumors were divided into two cohorts, that is, cohort 1 (60 cases) and cohort 2 (for validation; 60 cases). We analyzed the expression levels of these miRNAs in the first step (adenoma→IMC) and second step IMC→iCRC) of the adenoma–carcinoma sequence in both cohorts. Although no significant differences in the expression of any of the 13 miRNAs were found between adenomas and IMCs consistently in both cohorts, the expression levels of hsa‐miRNA‐125b‐5p, hsa‐miRNA‐143‐3p, and hsa‐miRNA‐206 were significantly upregulated in iCRC in both cohorts compared with those in IMC. The current results suggest that certain miRNAs, including hsa‐miRNA‐125b‐5p, hsa‐miRNA‐143‐3p and hsa‐miRNA‐206, are candidate markers that play critical roles in the progression of IMC to iCRC.

**Abbreviations**
AUC: area under the curve
CRC: colorectal cancer
miRNA: microRNA
PCR: polymerase chain reaction
ROC: receiver operating characteristic curve

## INTRODUCTION

Colorectal cancer (CRC) develops as a result of the pathological transformation of the normal colonic epithelium to invasive cancer from adenomatous lesions via intramucosal carcinoma according to the ‘adenoma–carcinoma’ sequence.^1^, ^2^ This multistep progression is accompanied by a number of characterized molecular mechanisms, such as genetic and epigenetic alterations including DNA methylation and microRNA (miRNA) functions.^3^, ^4^, ^5^ miRNAs are 19–23‐nucleotide noncoding endogenous single‐stranded RNAs that function as post‐transcriptional gene regulators by binding to their target messenger RNAs and have been implicated in tumor progression.^6^ Abnormal expression of miRNAs in colorectal neoplastic lesions has been investigated in CRC.^7^, ^8^, ^9^ Dysregulated miRNAs play an important role in the progression of CRC, but less is known about their role in colorectal adenomas.^10^, ^11^ Several studies have examined the expression of select miRNAs in colorectal adenomas, the precursor lesion of adenocarcinomas.^10^, ^11^ Changes in the expression patterns of miRNAs may be informative and highly significant in the colorectal adenoma–carcinoma sequence progression as well.^6^ However, abnormal expression of miRNAs contributing to the progression mechanism has been poorly investigated.^4^

The aim of this study was to identify the patterns of miRNA expression changes during neoplastic progression leading to the most frequent molecular subtype of CRC.

## MATERIALS AND METHODS

### Patients

A total of 120 patients comprising 40 with conventional adenomas, 20 with intramucosal adenocarcinomas and 60 with invasive CRC were enrolled in the present study. Conventional adenomas included tubular and tubulovillous adenomas that were histologically diagnosed according to the modified World Health Organization 2019 criteria.^12^, ^13^ The conventional adenomas were classified into low‐grade dysplasia, characterized by a uniform monolayer of columnar cells with basal nuclei showing minimal atypia, and high‐grade dysplasia, characterized by more frequent nuclear atypia, nuclear pleomorphism, nuclear enlargement and pseudo‐stratification without stromal invasion. Intramucosal carcinomas were characterized by marked cytological atypia and complex cribriform architecture with irregular branching, glandular anastomosis and neoplastic cells budding into the mucosa, which were considered to represent stromal invasion.^12^ Invasive CRC was defined as cancer invading past the mucosa with/without metastasis. Clinicopathological findings were recorded according to the General Rules for Management of the Japanese Colorectal Cancer Association.^12^

In the present study, the tumors were divided into cohort 1 (60 cases) and cohort 2, which was used for validation (60 cases). The clinicopathological findings of the cases in cohorts 1 and 2 are shown in Table 1.

**Table 1:** Clinicopathological findings of colorectal tumors

Cohort 1				
		Conventional adenomas (%)	Intramucosal carcinomas (%)	Invasive CRCs (%)
Total		20	10	30
Sex	Man	12 (60.0)	7 (70.0)	15 (50.0)
	Woman	8 (40.0)	3 (30.0)	15 (50.0)
Age (years)	Range (median)	51–81 (71)	58–76 (71)	39–84 (68)
Size (mm)	Range (median)	8–53 (17)	9–36 (26)	25–95 (44)
Location	Right	11 (55.5)	5 (50.0)	13 (43.3)
	Left	9 (45.0)	5 (50.0)	17 (56.7)
Macroscopic type	Pedunculated	15 (75.0)	7 (70.0)	
	Depressed	0	1 (10.0)	
	LST	5 (25.0)	2 (20.0)	
	Type1/2/3			30 (100)
Histological subtype	TA	13 (65.0)		
	TVA	7 (35.0)		
Histological grade	Low grade	16 (80.0)		
	High grade	4 (20.0)		
Differentiation	WDA		6 (60.0)	2 (6.7)
	MDA		3 (30.0)	27 (90.0)
	Pap		1 (10.0)	1 (3.3)
Tumor extension	pT1		10 (100)	0
	pT2		0	3 (10.0)
	pT3		0	21 (70.0)
	pT4		0	6 (20.0)

Cohort 2				
Total		20	10	30
Sex	Man	12 (60.0)	7 (70.0)	15 (50.0)
	Woman	8 (40.0)	3 (30.0)	15 (50.0)
Age (years)	Range (median)	42–83 (72)	57–80 (71)	53–90 (70)
Size (mm)	Range (median)	9–42 (20)	9–47 (25)	22–90 (50)
Location	Right	12 (60.0)	5 (50.0)	12 (40.0)
	Left	8 (40.0)	5 (50.0)	18 (60.0)
Macroscopic type	Pedunculated	14 (70.0)	5 (50.0)	
	Depressed	0	0	
	LST	6 (30.0)	5 (50.0)	
	Type1/2/3			30 (100)
Histological subtype	TA	13 (65.0)		
	TVA	7 (35.0)		
Histological grade	Low grade	16 (80.0)		
	High grade	4 (20.0)		
Differentiation	WDA		7 (70.0)	2 (6.7)
	MDA		2 (20.0)	26 (86.7)
	Pap		1 (10.0)	2 (6.7)
Tumor extension	pT1		10 (100)	0
	pT2		0	4 (13.3)
	pT3		0	23 (76.7)
	pT4		0	3 (10.0)

Abbreviations: CRC, colorectal cancer; LST, laterally spreading tumor; MDA, moderately differentiated adenocarcinoma; Pap, papillary adenocarcinoma; TA, tubular adenoma; TVA, tubulovillous adenoma; WDA, well differentiated adenocarcinoma.

### Crypt isolation technique for tumor cell isolation

Fresh tumor and normal tissue samples were obtained from endoscopic or surgical specimens. In the conventional adenomas and intramucosal carcinomas, isolated tumor glands were removed from the deeper tumor mucosa. In contrast, the invasive CRC samples were obtained primarily from the central area of the tumor and included the most invasive layer. Normal colonic mucosa samples were obtained from a distant site from the same patient (for surgically resected cancer specimens) or in the adjacent mucosa (for endoscopic specimens). If the corresponding isolated normal gland specimens could not be obtained from endoscopic mucosal resection, isolated normal gland samples were used as a normal control for miRNA analysis.

Crypt isolation from the tumor and normal mucosa was performed as described previously.^14^ Briefly, fresh mucosa and tumor tissues were minced with a razor into minute pieces and then incubated at 37°C for 30 min in calcium‐ and magnesium‐free Hanks’ balanced salt solution containing 30 mmol/L ethylenediaminetetraacetic acid. Next, the tissue was stirred in calcium‐ and magnesium‐free Hanks’ balanced salt solution for 30–40 min. The isolated crypts were immediately fixed in 70% ethanol and stored at 4°C until used for RNA extraction. The fixed isolated crypts were examined under a dissecting microscope (SZ60; Olympus, Tokyo, Japan). These crypts were then routinely processed for histopathological analysis to confirm their isolated nature based on morphology. No contamination (such as interstitial cells) was observed in any of the 120 samples.

### RNA isolation

miRNAs were extracted using the mirVana miRNA Isolation kit (Thermo Fisher Scientific, Inc., Waltham, MA, USA) according to the manufacturer's instructions. RNA quantity and quality were evaluated using the DU730 spectrophotometer (Beckman Coulter, Brea, CA, USA), and RNA integrity was determined by gel electrophoresis.

### Quantitative reverse‐transcription polymerase chain reaction analysis of miRNAs

The following miRNAs expressed in CRCs were evaluated in this study because of their implication in CRC based on previous studies: hsa‐miRNA‐19a‐3p,^15^ hsa‐miRNA‐21‐5p,^15^ hsa‐miRNA‐27a‐3p,^15^ hsa‐miRNA‐27b‐3p,^15^ hsa‐miRNA‐31‐5p,^15^ hsa‐miRNA‐34b‐3p,^15^ hsa‐miRNA‐125b‐5p,^16^ hsa‐miRNA‐143‐3p,^15^ miRNA‐191‐5p,^15^ hsa‐miRNA‐193b‐3p,^17^ hsa‐miRNA‐195‐5p,^15^ hsa‐miRNA‐206^18^ and hsa‐let‐7a‐5p.^15^ The primer sequences used are shown in Table S1.

The mature miRNAs were detected and quantified by quantitative reverse‐transcription polymerase chain reaction (PCR) in conjunction with TaqMan miRNA assays (Applied Biosystems, Foster City, CA, USA) as described previously.^19^ Triplicate RNA samples were used. RNA was reverse transcribed into complementary DNA (cDNA) using the TaqMan MicroRNA Reverse Transcription Kit (Applied Biosystems), and reactions were run on the Gene Amp PCR system 9700 thermal cycler (Applied Biosystems). Samples were incubated at 16°C for 30 min, followed by 42°C for 30 min and 85 °C for 5 min. We included a negative control lacking reverse transcriptase in each set of reactions. The reaction mixture (20 μL final volume) consisted of the cDNA product, TaqMan 2X Universal PCR Master Mix II and the appropriate 20X MicroRNA Assay Mix containing the specific probe targeting the miRNA of interest. PCR was performed using the StepOnePlus Real‐Time PCR System (Applied Biosystems) under the following reaction conditions: 10 min at 95 °C, followed by 40 cycles of 15 s at 95 °C and 60 s at 60 °C. Inter‐assay controls and calibrators were included in each 96‐well plate. All TaqMan assays were run in triplicate using the AB StepOnePlus Real‐Time PCR System. *RNU6B* (assay ID: 001093) was amplified as an endogenous control for normalization of the miRNA expression levels. Ct values were generated using StepOne Software v2.2.2 with the automatic baseline settings, and the 2^−ΔΔCt^ method was used to calculate the expression level of each miRNA in the tumor tissues relative (fold change) to the non‐tumor tissues.

### Statistical analysis

The expression levels of miRNAs (log_10_ ratio) from the three types of lesions (conventional adenomas, intramucosal carcinomas, and invasive CRC) were analyzed using the JMP 10.0 software package (SAS Institute, Cary, NC, USA) with Bonferroni corrections. Clinicopathological variables (sex, location, macroscopic type, histological subtype, histological grade, differentiation and tumor extension) were analyzed using Fisher's exact test.

If statistical differences among the three lesion types were detected, comparisons between two groups were performed using Fisher's exact test. Differences in age and tumor size between two groups were evaluated using the Mann–Whitney *U*‐test. Differences with a *P* value less than 0.05 were considered significant. Comparisons between matched sample pairs were conducted using McNemar's test.

Cut‐off expression levels for each miRNA were determined using receiver operating characteristic (ROC) analysis. For each cut‐off expression level, the weighted mean sensitivity and specificity values for differentiating between lesion types were plotted to generate a ROC curve. The expression level closest in distance to the point on the curve with both the maximum sensitivity and specificity was selected as the cut‐off, representing the expression level that correctly classified the greatest number of tumors with or without downregulation of that miRNA. The area under the ROC curve (AUC) was then calculated. These analyses were also conducted using JMP 10.0 software.

## RESULTS

To confirm and validate the differential expression of each miRNA examined in the current study, we first measured miRNA expression levels in isolated glands from 120 colorectal tumor specimens and compared the results with corresponding paired normal samples.

**Figure 1:**
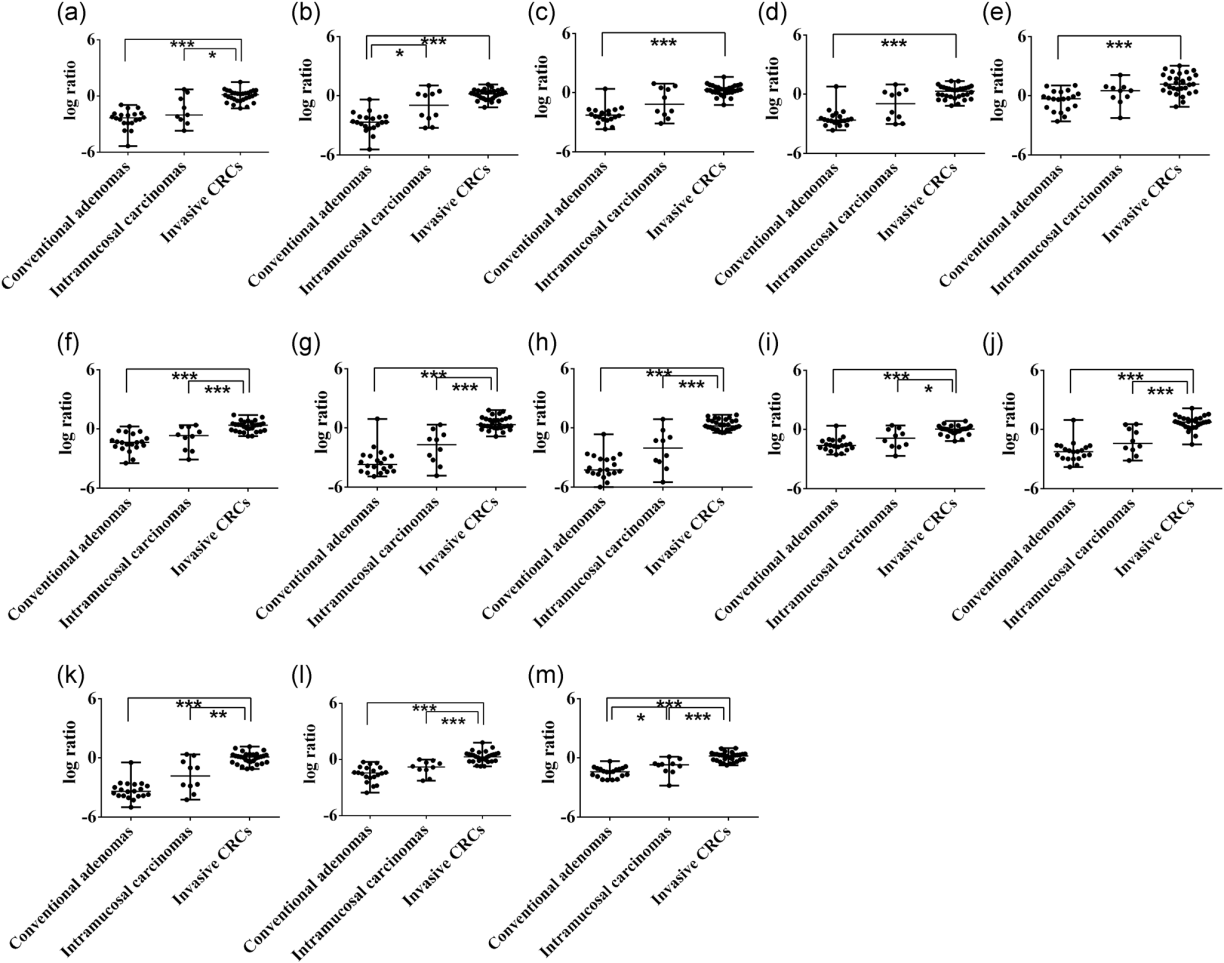
Expression of the microRNAs (miRNAs) examined in colorectal tumors in cohort 1. (**a**) hsa‐miRNA‐19a‐3p; (**b**) hsa‐miRNA‐21‐5p; (**c**) hsa‐miRNA‐27a‐3p; (**d**) hsa‐miRNA‐27b‐3p; (**e**) hsa‐miRNA‐31‐5p; (**f**) hsa‐miRNA‐34b‐3p; (**g**) hsa‐miRNA‐125b‐5p; (**h**) hsa‐miRNA‐143‐3p; (**i**) hsa‐miRNA‐191‐5p; (**j**) hsa‐miRNA‐193b‐3p; (**k**) hsa‐miRNA‐195‐5p; (**l**) hsa‐miRNA‐206; (**m**) hsa‐let‐7a‐5p. *, *P* < 0.05; **, *P* < 0.01; ***, *P* < 0.001.

**Figure 2:**
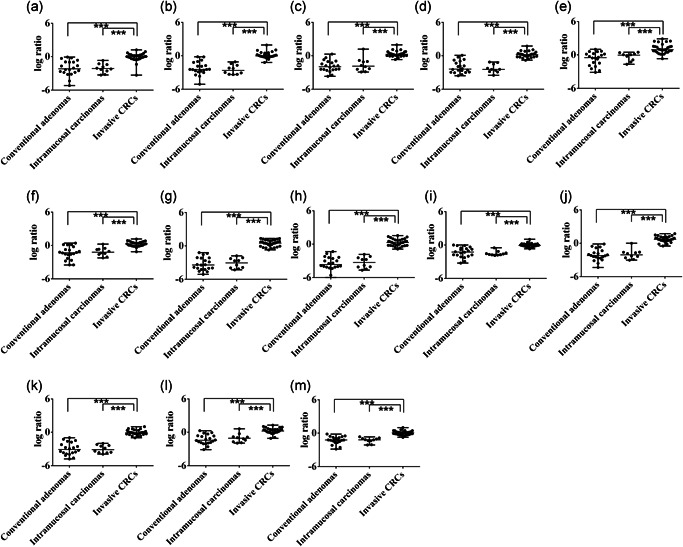
Expression of the microRNAs (miRNAs) examined in colorectal tumors in cohort 2. (**a**) hsa‐miRNA‐19a‐3p; (**b**) hsa‐miRNA‐21‐5p; (c) hsa‐miRNA‐27a‐3p; (**d**) hsa‐miRNA‐27b‐3p; (**e**) hsa‐miRNA‐31‐5p; (**f**) hsa‐miRNA‐34b‐3p; (**g**) hsa‐miRNA‐125b‐5p; (**h**) hsa‐miRNA‐143‐3p; (**i**) hsa‐miRNA‐191‐5p; (**j**) hsa‐miRNA‐193b‐3p; (**k**) hsa‐miRNA‐195‐5p; (**l**) hsa‐miRNA‐206; (**m**) hsa‐let‐7a‐5p. *, *P* < 0.05; **, *P* < 0.01; ***, *P* < 0.001.

1. Differences in miRNA dysregulation among each lesion type in cohort 1
  In cohort 1, there were statistically significant differences in the downregulation of hsa‐miRNA‐19a‐3p, hsa‐miRNA‐34b‐3p, hsa‐miRNA‐125b‐5p, hsa‐miRNA‐143‐3p, miRNA‐191‐5p, hsa‐miRNA‐193b‐3p, hsa‐miRNA‐195‐5p and hsa‐miRNA‐206 between conventional adenomas and invasive CRCs and between intramucosal carcinomas and invasive CRCs, compared with specimens isolated from normal glands. The median expression levels of hsa‐miRNA‐27a‐3p, hsa‐miRNA‐27b‐3p and hsa‐miRNA‐31‐5p were significantly higher in invasive CRC than in conventional adenomas, and the median level of hsa‐miRNA‐21‐5p was significantly higher in intramucosal carcinomas and invasive CRC compared with conventional adenomas. Finally, there was a significant difference in the median level of hsa‐let‐7a‐5p between conventional adenomas and intramucosal carcinomas, between intramucosal carcinomas and invasive CRC and between conventional adenomas and invasive CRC, compared with specimens isolated from normal glands. These data are shown in Fig. 1.
2. Differences in miRNA dysregulation among each lesion type in cohort 2 (validation cohort)
  Statistically significant differences were found in the downregulation in colorectal lesions of all 13 miRNAs between conventional adenomas and invasive CRC and between intramucosal carcinomas and invasive CRC, compared with specimens isolated from normal glands. In the miRNAs we examined, there were few overlapping cases with regard to the distributions of specific miRNAs, including hsa‐miRNA‐125b‐5p and hsa‐miRNA‐143‐3p, between intramucosal carcinoma and invasive cancer. The data are depicted in Fig. 2.
  First, we attempted to determine the cut‐off value of the expression level for each miRNA to differentiate intramucosal carcinoma from adenoma and predict the invasion of invasive CRC from intramucosal carcinoma in cohort 1. Next, we validated whether the obtained miRNAs could be effective for the differential diagnosis of intramucosal carcinoma from adenoma and prediction of invasion of intramucosal carcinoma to invasive CRC using the cut‐off value.
3. miRNA expression level differences between conventional adenomas and intramucosal carcinomas
  3. Cut‐off expression levels of each miRNA for differentiating conventional adenomas from intramucosal carcinomas in cohort 1.
    Receiver operating characteristic curve (ROC) analysis was used to determine the cut‐off expression level of each examined miRNA that best differentiated between conventional adenomas and intramucosal carcinomas. The AUC and optimal cut‐off expression level of each miRNA are shown in Supplementary Fig. S1.
  4. miRNAs differentiating conventional adenomas from intramucosal carcinomas, determined using statistical indicators
  The cut‐off value, AUC, sensitivity, specificity, positive and negative predictive values, and positive and negative likelihood ratios of discriminating between the colorectal lesion types for each examined miRNA are shown in Table 2. To select the best miRNAs for differentiating intramucosal carcinomas from conventional adenomas, we used cut‐off criteria of a positive likelihood ratio >2 and negative likelihood ratio <0.5 (mild decision criteria), according to a previous study.^20^ hsa‐miRNA‐21b‐5p, hsa‐miRNA‐125b‐5p, hsa‐miRNA‐193b‐3p and hsa‐let‐7a‐5p met these criteria in cohort 1, but not in cohort 2 (validation cohort).
4. miRNA expression level differences between intramucosal carcinoma and invasive CRC
  4. Cut‐off expression levels of each miRNA for differentiating intramucosal carcinomas from invasive CRC
    ROC analysis was used to determine the cut‐off expression level of each miRNA that best differentiated invasive CRCs from intramucosal carcinomas. The AUC and optimal cut‐off expression level of each examined miRNA are shown in Fig. S2.
  5. miRNAs differentiating intramucosal carcinomas from invasive CRC, determined using statistical indicators
  The cut‐off value, AUC, sensitivity, specificity, positive and negative predictive values, and positive and negative likelihood ratios of discriminating between intramucosal carcinomas and invasive CRC for each miRNA are shown in Table 3. To identify the appropriate miRNAs for differentiating invasive CRCs from intramucosal carcinomas, we used cut‐off criteria of a positive likelihood ratio >2 and negative likelihood ratio <0.5. Of the 13 miRNAs, 11 met these criteria in cohort 1: hsa‐miRNA‐19a‐3p, hsa‐miRNA‐27a‐3p, hsa‐miRNA‐31‐5p, hsa‐miRNA‐34b‐3p, hsa‐miRNA‐125b‐5p, hsa‐miRNA‐143‐3p, miRNA‐191‐5p, hsa‐miRNA‐193b‐3p, hsa‐miRNA‐195‐5p, hsa‐miRNA‐206 and hsa‐let‐7a‐5p. Therefore, to narrow the pool of candidate miRNAs differentiating intramucosal carcinomas from invasive CRCs, we set more stringent criteria of a positive likelihood ratio >5 and negative likelihood ratio <0.2, according to a previous report.^20^ hsa‐miRNA‐125b‐5p, hsa‐miRNA‐143‐3p and hsa‐miRNA‐206 met these thresholds and thus were selected as strong markers differentiating intramucosal carcinoma from invasive CRC based on their expression levels. These miRNAs also met our criteria in cohort 2 (validation cohort).

**Table 2:** Cut‐off microRNA expression levels, with the area under the curve, sensitivity, specificity, positive predictive value, negative predictive value, positive likelihood, and negative likelihood for discriminating between conventional adenomas and intramucosal carcinomas

Cohort 1								
Micro RNA	Cut‐off	AUC	Se (%)	Sp (%)	PPV (%)	NPV (%)	PLH	NLH
hsa‐miRNA‐19a‐3p	−1.25762	0.656	50.0	90.0	71.4	78.3	5	0.56
hsa‐miRNA‐21b‐5p	−1.96679	0.780	60.0	85.0	66.7	81.0	4	0.47
hsa‐miRNA‐27a‐3p	−0.492	0.720	50.0	95.0	83.3	79.2	10	0.53
hsa‐miRNA‐27b‐3p	−1.15989	0.735	50.0	95.0	83.3	79.2	10	0.53
hsa‐miRNA‐31‐5p	0.51019	0.700	50.0	85.0	62.5	77.3	3.33	0.59
hsa‐miRNA‐34b‐3p	−0.87164	0.635	60.0	75.0	54.5	78.9	2.4	0.53
hsa‐miRNA‐125b‐5p	−2.17907	0.755	60.0	90.0	75.0	81.8	6	0.44
hsa‐miRNA‐143‐3p	−3.41665	0.765	70.0	65.0	50.0	81.3	2	0.46
hsa‐miRNA‐191‐5p	−0.62156	0.695	40.0	95.0	80.0	76.0	8	0.63
hsa‐miRNA‐193b‐3p	−2.05025	0.725	80.0	65.0	53.3	86.7	2.29	0.31
hsa‐miRNA‐195‐5p	−2.83956	0.765	80.0	75.0	61.5	88.2	3.2	0.27
hsa‐miRNA‐206	−1.10592	0.730	80.0	70.0	57.1	87.5	2.67	0.29
hsa‐let‐7a‐5p	−0.82013	0.785	60.0	95.0	85.7	82.6	12	0.42

Cohort 2								
hsa‐miRNA‐19a‐3p	−1.25762	0.530	10.0	65.0	12.5	59.1	0.29	1.39
hsa‐miRNA‐21b‐5p	−1.96679	0.570	30.0	60.0	27.3	63.2	0.75	1.17
hsa‐miRNA‐27a‐3p	−0.492	0.555	10.0	90.0	33.3	66.7	1	1
hsa‐miRNA‐27b‐3p	−1.15989	0.525	10.0	75.0	16.7	62.5	0.40	1.20
hsa‐miRNA‐31‐5p	0.51019	0.580	0	80.0	0	61.5	0	1.25
hsa‐miRNA‐34b‐3p	−0.87164	0.550	40.0	65.0	36.4	68.4	1.14	0.92
hsa‐miRNA‐125b‐5p	−2.17907	0.540	20.0	70.0	25.0	63.6	0.67	1.14
hsa‐miRNA‐143‐3p	−3.41665	0.525	50.0	55.0	35.7	68.8	1.11	0.91
hsa‐miRNA‐191‐5p	−0.62156	0.575	10.0	75.0	16.7	62.5	0.40	1.20
hsa‐miRNA‐193b‐3p	−2.05025	0.545	50.0	60.0	38.5	70.1	1.25	0.83
hsa‐miRNA‐195‐5p	−2.83956	0.495	40.0	55.0	30.8	64.7	0.89	1.09
hsa‐miRNA‐206	−1.10592	0.575	50.0	65.0	41.7	72.2	1.43	0.77
hsa‐let‐7a‐5p	−0.82013	0.493	10.0	70.0	14.3	60.9	0.33	1.29

Abbreviations: AUC, area under the curve; CRC, colorectal cancer; NLH, negative likelihood; NPV, negative predictive value; PLH, positive likelihood; PPV, positive predictive value; Se, sensitivity; Sp, specificity.

**Table 3:** Cut‐off microRNA expression levels, with the area under the curve, sensitivity, specificity, positive predictive value, negative predictive value, positive likelihood, and negative likelihood for discriminating between intramucosal carcinomas and invasive CRCs

Cohort 1								
Micro RNA	Cut‐off	AUC	Se (%)	Sp (%)	PPV (%)	NPV (%)	PLH	NLH
hsa‐miRNA‐19a‐3p	−1.16787	0.774	93.3	60.0	87.5	75.0	2.33	0.11
hsa‐miRNA‐21b‐5p	−1.16615	0.690	96.7	50.0	85.3	83.3	1.93	0.07
hsa‐miRNA‐27a‐3p	−0.39402	0.700	90.0	60.0	87.1	66.7	2.25	0.17
hsa‐miRNA‐27b‐3p	−0.0888	0.720	66.7	60.0	83.3	37.5	1.67	0.56
hsa‐miRNA‐31‐5p	1.12704	0.733	56.7	90.0	94.4	40.9	5.67	0.48
hsa‐miRNA‐34b‐3p	−0.37431	0.839	93.3	60.0	87.5	75.0	2.33	0.11
hsa‐miRNA‐125b‐5p	−0.46042	0.928	100	90.0	96.8	100	10	0
hsa‐miRNA‐143‐3p	−0.45457	0.907	100	90.0	96.8	100	10	0
hsa‐miRNA‐191‐5p	−0.36071	0.757	80.0	70.0	88.9	53.8	2.67	0.29
hsa‐miRNA‐193b‐3p	0.12956	0.914	86.7	80.0	92.9	66.7	4.33	0.17
hsa‐miRNA‐195‐5p	−0.89939	0.813	93.3	70.0	90.3	77.8	3.11	0.01
hsa‐miRNA‐206	−0.25065	0.917	90.0	90.0	96.4	75.0	9	0.11
hsa‐let‐7a‐5p	−0.55017	0.897	93.3	80.0	93.3	80.0	4.67	0.08

Cohort 2								
hsa‐miRNA‐19a‐3p	−1.16787	0.960	93.3	90.0	96.6	81.8	9.33	0.07
hsa‐miRNA‐21b‐5p	−1.16615	0.997	96.7	90.0	96.7	90.0	9.67	0.04
hsa‐miRNA‐27a‐3p	−0.39402	0.903	96.7	90.0	96.7	90.0	9.67	0.04
hsa‐miRNA‐27b‐3p	−0.0888	1.0	76.7	100	100	58.8	∞	0.23
hsa‐miRNA‐31‐5p	1.12704	0.935	40.0	100	100	35.7	∞	0.60
hsa‐miRNA‐34b‐3p	−0.37431	0.938	93.3	90.0	96.6	81.8	9.33	0.07
hsa‐miRNA‐125b‐5p	−0.46042	1.0	93.3	100	100	83.3	∞	0.07
hsa‐miRNA‐143‐3p	−0.45457	1.0	100	100	100	100	∞	0
hsa‐miRNA‐191‐5p	−0.36071	0.993	86.7	100	100	71.4	∞	0.13
hsa‐miRNA‐193b‐3p	0.12956	0.990	90.0	100	100	76.9	∞	0.10
hsa‐miRNA‐195‐5p	−0.89939	1.0	96.7	100	100	90.9	∞	0.03
hsa‐miRNA‐206	−0.25065	0.897	90.0	90.0	96.4	75.0	9	0.11
hsa‐let‐7a‐5p	−0.55017	0.997	96.7	100	100	90.9	∞	0.03

Abbreviations: AUC, area under the curve; CRC, colorectal cancer; NLH, negative likelihood; NPV, negative predictive value; PLH, positive likelihood; PPV, positive predictive value; Se, sensitivity; Sp, specificity.

## DISCUSSION

Analyses of miRNA expression in colorectal adenomas have been reported infrequently. In previous studies, specific miRNAs, including hsa‐miRNA‐17‐92 cluster,^21^, ^22^ hsa‐miRNA‐21,^21^, ^23^ hsa‐miRNA‐135a/b,^21^, ^24^ hsa‐miRNA137,^21^, ^25^ hsa‐miRNA‐143 and hsa‐miRNA‐145,^21^, ^26^ were found to have altered expression in colorectal adenomas. In a previous global evaluation of the expression of 735 miRNAs, 31 had a fold change of greater than or equal to 2 in adenomas compared with normal mucosa specimens, including hsa‐miRNA‐135a, hsa‐miRNA‐135b, and hsa‐miRNA‐137.^27^ In another genome‐wide analysis, the expression levels of hsa‐miRNA‐19a, hsa‐miRNA‐20a, hsa‐miRNA‐21, hsa‐miRNA‐92a and hsa‐miRNA‐135b were altered among 866 hsa‐miRNAs evaluated in colorectal tumors.^21^ However, discordant results regarding expression of the examined hsa‐miRNAs were reported among the two studies.^21^, ^27^ Differences in miRNA expression among previous studies might be due to methodologic differences in terms of the analysis platform, miRNA annotation and criteria for differential expression, as well as study design differences in terms of patient population, tissue preparation (frozen or formalin‐fixed paraffin‐embedded) and sample size.^21^ In the present study, although we examined a limited number of miRNAs, we believe that these miRNAs are important markers that play major roles in the progression of CRC via the adenoma–carcinoma sequence based on our current results.

Colorectal tumorigenesis is thought to be a multistep process in which genetic alterations accumulate, ultimately producing an aggressive phenotype (adenoma–carcinoma sequence).^1^, ^2^ A model was proposed to explain the molecular mechanism underlying colorectal neoplasia development that includes several key molecular events.^1^, ^2^ We identified miRNAs that were commonly downregulated in conventional adenomas and intramucosal carcinomas compared with invasive CRCs in both cohorts 1 and 2. This finding indicates that miRNA expression is altered in the progression from conventional adenoma to invasive CRC, via intramucosal carcinoma, suggesting that the target genes regulated by miRNAs are also altered during tumorigenesis, given that one specific miRNA may have several different target genes. Interestingly, all examined miRNAs in the adenomas and intramucosal carcinomas were downregulated in both cohorts compared with samples isolated from normal glands, suggesting that these miRNAs are tumor suppressors that when downregulated allow expression of target oncogenes.

According to the adenoma–carcinoma sequence, colorectal carcinogenesis involves two steps: the transition from conventional adenoma to intramucosal carcinoma and the transition from intramucosal carcinoma to invasive CRC. In the present study, none of the 13 miRNAs examined were associated with the transformation from adenoma to intramucosal carcinoma. However, hsa‐miRNA‐125b‐5p, hsa‐miRNA‐143‐3p and hsa‐miRNA‐206 were closely associated with the progression from intramucosal carcinoma to invasive CRC. Ozawa *et al*.^17^ proposed that five miRNAs (miRNA‐32, miRNA‐181b‐1, miRNA‐193b, miRNA‐195 and miRNA‐411) may be useful predictors of the risk of submucosal invasion in CRC. Our results suggest that three additional miRNAs (hsa‐miRNA‐125b‐5p, hsa‐miRNA‐143‐3p and hsa‐miRNA‐206) might also be helpful in predicting the risk of invasion from the mucosa to submucosa.

A previous study showed that miRNA‐125b‐5p suppressed cell invasion and migration by targeting breast‐cancer metastasis suppressor 1 (BRMS1), which was found to inhibit cancer metastasis in gastric cancer cells.^28^ However, another study demonstrated that expression of miRNA‐125b‐5p is correlated with a poor prognosis in hepatocellular carcinoma patients by targeting thioredoxin‐1 (TXNRD1), a key molecule associated with intracellular redox homeostasis that promotes energy and carbohydrate metabolism.^29^ In this study, miRNA‐125b‐5p was upregulated in invasive CRC, compared with samples isolated from normal samples, although this finding was not consistent with previous studies.^28^, ^29^

Regarding miRNA‐143‐3p, we demonstrated that its expression was correlated with invasive CRCs compared with intramucosal carcinomas. A previous study revealed that miRNA‐143‐3p overexpression suppressed cell proliferation, migration and invasion in CRC.^29^ Another study indicated that miRNA‐143‐3p inhibits catenin delta 1 (CTNND1) in CRC, and that forced expression of CTNND1 induced cell proliferation, migration and invasion in CRC, whereas CTNND1 silencing had the opposite effects.^30^ Thus, miRNA‐143‐3p may act as a tumor suppressor by negatively regulating CTNND1, which is considered an oncogene.^30^ A recent study showed QKI‐5, a key post‐transcriptional regulator that acts as a tumor suppressor gene in CRC, was identified as a direct target of miRNA‐143‐3p in that study.^31^

Finally, miRNA‐206 was suggested as a candidate marker of CRC invasion in the present study. The expression of miRNA‐206 was significantly reduced in laryngeal cancer tissues compared with paired adjacent non‐neoplastic tissues in a previous study,^32^ suggesting that loss of miRNA‐206 may be correlated with laryngeal carcinogenesis. Moreover, the miRNA‐206 level was inversely correlated with clinicopathological findings, including poor differentiation, tumor cell grade, lymph node metastasis and advanced clinical stage^32^, ^33^ and thus downregulated miRNA‐206 may play an important role in the progression of laryngeal cancer.^32^ A recent study showed that miRNA‐206 inhibits PGE2‐induced CRC cell proliferation, migration and invasion by targeting transmembrane 4 L six family member 1 (TM4SF1), which is associated with regulation of cell development, activation, growth and motility.^33^ Those findings may not support our result of significantly higher miRNA‐206 expression in invasive CRCs than intramucosal carcinomas and conventional adenomas, and the reason for the discrepancy is unknown. It is possible that upregulation of miRNAs such as miRNA‐125b‐5p, miRNA‐143‐3p and miRNA‐206 may result in a tumor oncogenic effect via targeting of unknown oncogenes. Discovery of such target genes will be necessary to elucidate the present results.

Analysis of the heterogeneous expression of miRNAs occurring in colorectal cancer is important in colorectal carcinogenesis.^34^ However, we could not analyze the heterogeneity of expression of the examined miRNAs because multiple sampling was not performed in the current study. However, the expression of specific miRNAs, including hsa‐miRNA‐125b‐5p, hsa‐miRNA‐143‐3p and hsa‐miRNA‐206, may be associated with intratumoral heterogeneity, which promotes tumor invasiveness, metastatic ability, and drug sensitivity inherent to cancer cells.

There are some limitations to this study. First, the number of miRNAs examined was small. Comprehensive analyses may be necessary to search for novel candidate markers that can aid the diagnosis of colorectal tumors and predict the invasiveness of CRC.^21^, ^35^ However, we attempted to examine colorectal neoplastic lesions including conventional adenomas, intramucosal carcinomas and invasive CRC using a select pool of markers that have reliably been reported to contribute to colorectal carcinogenesis. We believe that despite the limited number, these miRNAs are useful candidate markers potentially predicting cancer invasion. Second, in situ hybridization may be required to confirm whether miRNA expression may be derived from isolated tumor glands.^36^ However, we believe that the miRNAs we examined originated from isolated tumor glands because we are technically skilled at crypt isolation and could therefore accurately obtain well‐isolated tumor glands without other tissues.^14^, ^19^ Finally, previous reports have shown that there are two types of cancer cells, that is, low and high grades, in intramucosal carcinoma. However, the crypt isolation method used in the current study could not differentiate low‐grade cancer glands from high‐grade cancer glands. Therefore, we could not examine the expression of the miRNAs assessed in this study based on histological differences, such as low‐ and high‐grade cancer in intramucosal carcinoma.

In conclusion, we identified three specific miRNAs closely associated with cancer invasion in both cohorts. However, we failed to clarify the association of the specific miRNAs with the progression from conventional adenoma to intramucosal carcinoma. Understanding the nature of miRNA altered expression is essential for the effective use of miRNAs as biomarkers and therapeutic targets because miRNA differential expression is linked to steps within the adenoma–carcinoma sequence.

## DISCLOSURE STATEMENT

None declared.

## AUTHOR CONTRIBUTIONS

ME performed all data collection and analyses. TS, who is the corresponding author, contributed to the preparation of the manuscript, including all aspects of the data collection and analysis. MO, AS and YF generated the figures and tables and performed the statistical analyses. HS performed the molecular analyses. YT, KO, AS and TM provided clinical support during the preparation of the manuscript.

## ETHICAL APPROVAL AND CONSENT TO PARTICIPATE

Informed consent was obtained from each patient according to institutional guidelines, and the research protocols were approved by the ethics committee of Iwate Medical University Hospital (reference number: HG2018‐530).

## CONSENT FOR PUBLICATION

We guarantee that (i) the work is original; (ii) the work has not been and will not be published in whole, or in part, in any other journal; and (iii) all of the authors have agreed to the contents of the manuscript in its submitted form.

## SUPPORTING INFORMATION

Supplementary Figure S1 ROC analyses of miRNA expression levels for differentiating between adenomas and intramucosal carcinomas in cohort 1. (**a**) hsa‐miRNA‐19a‐3p; (**b**) hsa‐miRNA‐21‐5p; (**c**) hsa‐miRNA‐27a‐3p; (**d**) hsa‐miRNA‐27b‐3p; (**e**) hsa‐miRNA‐31‐5p; (**f**) hsa‐miRNA‐34b‐3p; (**g**) hsa‐miRNA‐125b‐5p; (**h**) hsa‐miRNA‐143‐3p; (**i**) hsa‐miRNA‐191‐5p; (**j**) hsa‐miRNA‐193b‐3p; (**k**) hsa‐miRNA‐195‐5p; (**l**) hsa‐miRNA‐206; (**m**) hsa‐let‐7a‐5p.

Supplementary Figure S2 ROC analyses of miRNA expression levels for differentiating between intramucosal carcinomas and invasive CRCs in cohort 1. (**a**) hsa‐miRNA‐19a‐3p; (**b**) hsa‐miRNA‐21‐5p; (**c**) hsa‐miRNA‐27a‐3p; (**d**) hsa‐miRNA‐27b‐3p; (**e**) hsa‐miRNA‐31‐5p; (**f**) hsa‐miRNA‐34b‐3p; (**g**) hsa‐miRNA‐125b‐5p; (**h**) hsa‐miRNA‐143‐3p; (**i**) hsa‐miRNA‐191‐5p; (**j**), hsa‐miRNA‐193b‐3p; (**k**) hsa‐miRNA‐195‐5p; (**l**) hsa‐miRNA‐206; (**m**) hsa‐let‐7a‐5p.

Supplementary Table S1. List of primers used for quantitative reverse‐transcription PCR

## Supporting information

Additional Supporting Information may be found in the online version of this article at the publisher's website.

Supporting information.

Supporting information.

Supporting information.

Supporting information.
