# Supplementary figures and images for: Dysregulation of microRNA expression during the progression of colorectal tumors
Source: Pathol Int. 2020 Jun 26;70(9):633–43. doi: 10.1111/pin.12975 (PMC7540039; doi:10.1111/pin.12975)

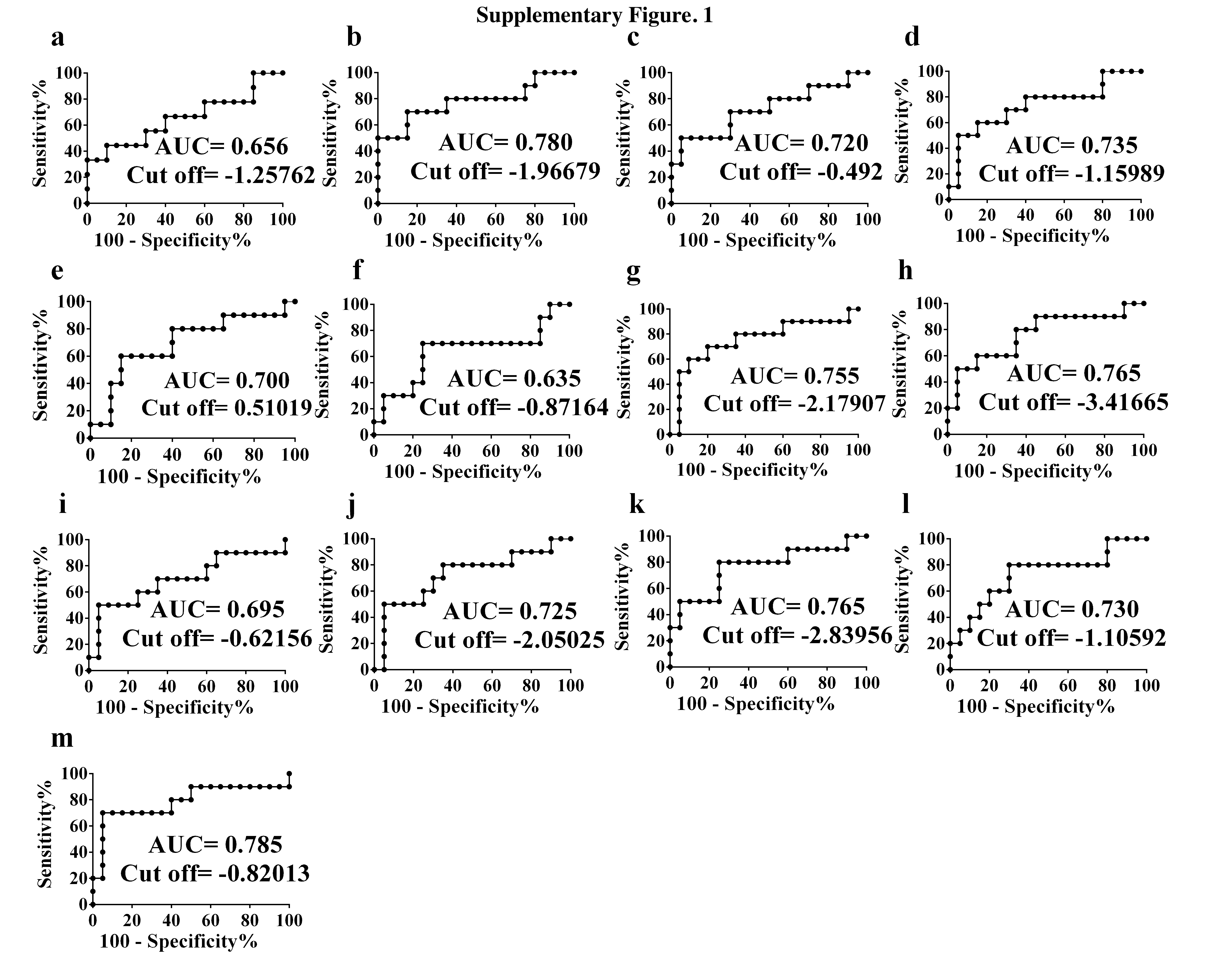

Supplement: Supplementary file 1 — Supporting information. [file PIN-70-633-s001.tif]

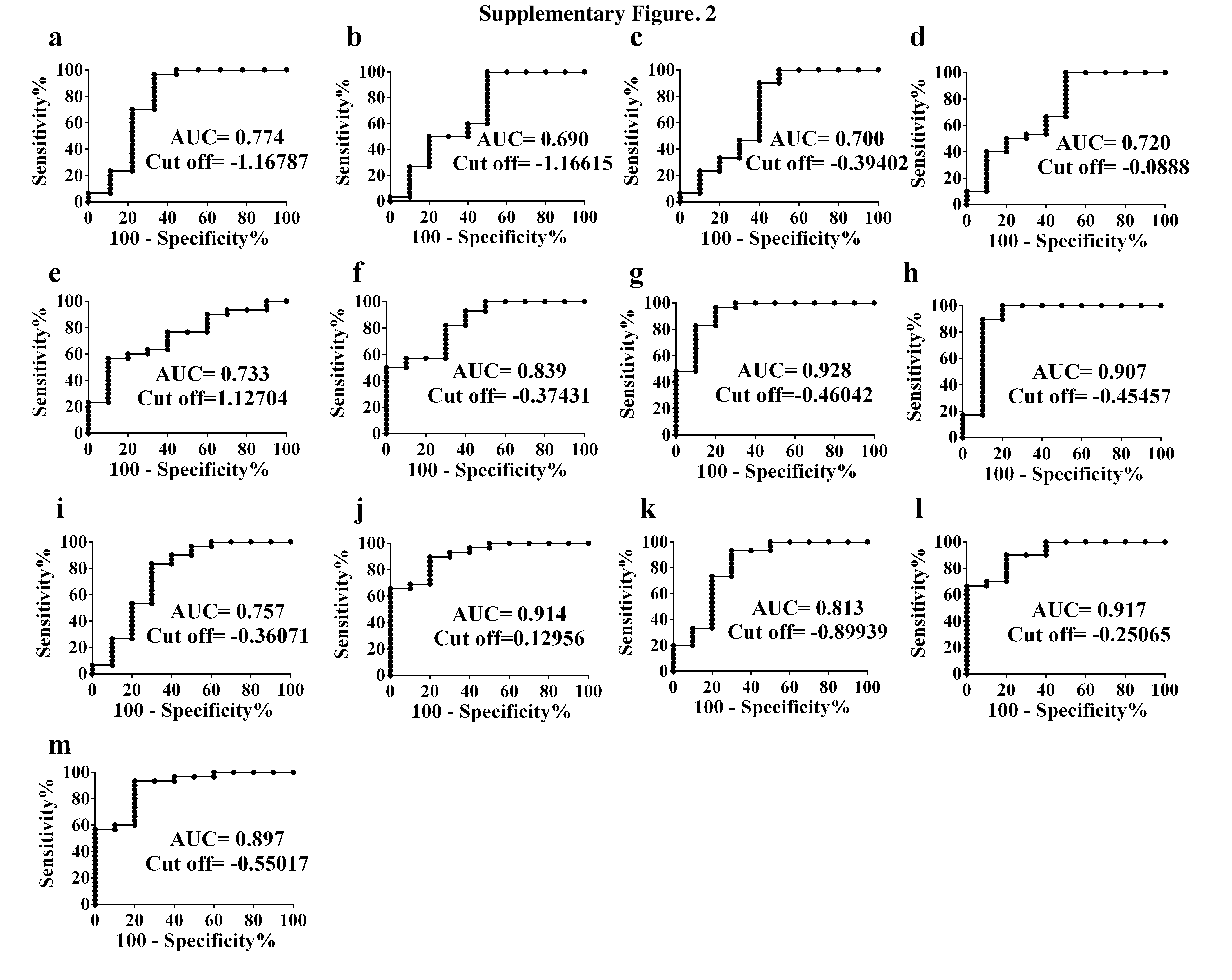

Supplement: Supplementary file 2 — Supporting information. [file PIN-70-633-s002.tif]
