# Supplementary material for: Dysregulation of microRNA expression during the progression of colorectal tumors
Source: Pathol Int. 2020 Jun 26;70(9):633–43. doi: 10.1111/pin.12975 (PMC7540039; doi:10.1111/pin.12975)
Supplement: Supplementary file 3 — Supporting information. [file PIN-70-633-s003.docx]

**Supplementary Table 1.** List of primers used for quantitative reverse-transcription PCR

| Micro RNA | Assay ID | Sequence (5’-3’) |
| --- | --- | --- |
| hsa-miRNA-19a-3p | 000395 | UGUGCAAAUCUAUGCAAAACUGA |
| hsa-miRNA-21b-5p | 000397 | UAGCUUAUCAGACUGAUGUUGA |
| hsa-miRNA-27a-3p | 000408 | UUCACAGUGGCUAAGUUCCGC |
| hsa-miRNA-27b-3p | 000409 | UUCACAGUGGCUAAGUUCUGC |
| hsa-miRNA-31-5p | 002279 | AGGCAAGAUGCUGGCAUAGCU |
| hsa-miRNA-34b-3p | 002102 | CAAUCACUAACUCCACUGCCAU |
| hsa-miRNA-125b-5p | 000449 | UCCCUGAGACCCUAACUUGUGA |
| hsa-miRNA-143-3p | 002249 | UGAGAUGAAGCACUGUAGCUC |
| hsa-miRNA-191-5p | 002299 | CAACGGAAUCCCAAAAGCAGCUG |
| hsa-miRNA-193b-3p | 002367 | AACUGGCCCUCAAAGUCCCGCU |
| hsa-miRNA-195-5p | 000494 | UAGCAGCACAGAAAUAUUGGC |
| hsa-miRNA-206 | 000510 | UGGAAUGUAAGGAAGUGUGUGG |
| hsa-let-7a-5p | 000377 | UGAGGUAGUAGGUUGUAUAGUU |
