# Supplementary material for: Dysregulation of microRNA expression during the progression of colorectal tumors
Source: Pathol Int. 2020 Jun 26;70(9):633–43. doi: 10.1111/pin.12975 (PMC7540039; doi:10.1111/pin.12975)
Supplement: Supplementary file 4 — Supporting information. [file PIN-70-633-s004.docx]

**List of Supplementary Material**

Supplementary Fig. 1 ROC analyses of miRNA expression levels for differentiating between adenomas and intramucosal carcinomas in cohort 1.

Supplementary Fig. 2 ROC analyses of miRNA expression levels for differentiating between intramucosal carcinomas and invasive CRCs in cohort 1.

Supplementary Table 1. List of primers used for quantitative reverse-transcription PCR
